# Retrospective cohort evaluation study in terms of cardiovascular and metabolic diseases in chronic hepatitis B patients

**DOI:** 10.3389/fendo.2024.1426196

**Published:** 2024-10-03

**Authors:** Aysun Yakut

**Affiliations:** Department of Gastroenterology, İstanbul Medipol University Sefakoy Health Practice Research Center, Istanbul, Türkiye

**Keywords:** insulin resistance, cardiovascular diseases, chronic hepatitis B, nucleotide analogues, TyG index, TG\HDL-C ratio, TC\HDL-C ratio, FIB-4 score

## Abstract

**Background:**

Chronic hepatitis B (CHB) and nucleotide analogues [entecavir (ETV) and tenofovir disoproxil fumarate (TDF)] used in its treatment have been shown to affect metabolic parameters in many studies. In this study, we aimed to investigate the effects of metabolic events associated with CHB and nucleotide analogues (NAs) used in CHB treatment on ischemic heart diseases (IHD) and cardiovascular diseases (CVD).

**Methods:**

This retrospective study was conducted between June 2022 and January 2024 with a total of 241 patients diagnosed with non-cirrhotic CHB in the gastroenterology outpatient clinic, 96 of whom did not receive hepatitis B treatment, 110 of whom received TDF, and 35 of whom received ETV treatment. Patients were evaluated in terms of metabolic, CVD, and hepatology depending on whether they received antiviral treatment or not. In our study, the triglyceride-glucose (TyG) index and triglyceride-to-high-density lipoprotein cholesterol ratio (TG/HDL-C) were calculated in patients to evaluate potential risk factors for CVD. Again, while the total cholesterol-to-HDL-C ratio (TC/HDL-C), which is associated with CVD\IHD, was evaluated, the ‘4-factor fibrosis index’ (FIB-4) score, which is a non-invasive indicator of liver fibrosis, was also evaluated.

**Results:**

Diabetes mellitus (DM), fasting blood sugar (FBS), oral antidiabetic drug (OAD) usage rate, and insulin usage rate were high in patients receiving ETV treatment. The TyG index of patients receiving ETV was higher than patients in the other group (p = 0.035; p<0.05). It was determined that the probability of detecting ETV treatment in patients with a TG/HDL-C ratio of ≥1.82 cut-off value was 4.250 times higher. The odds ratio for TG/HDL-C measurements was 4.250 (95% CI: 1.384–13.054). FIB-4 score, which is a non-invasive indicator of liver fibrosis, was found to be higher in patients receiving ETV than in other groups.

**Conclusion:**

In patients with CHB, a relationship was observed between markers used to predict CVD risk, such as the TyG index and TG/HDL-C ratio. The group with high levels of these two markers and a high potential for developing CVD was patients receiving ETV treatment. In this first study in the literature showing the relationship between CHB and CVD, we found that the relative risk of CVD was increased in patients using ETV.

## 1 Introduction

The leading cause of premature death worldwide is cardiovascular disease (CVD) (1). Recent studies have shown that insulin resistance (IR) increases the risk of CVD independently of traditional risk factors such as age, smoking, and hypertension (2). There is an increased risk of metabolic syndrome and CVD in euglycemic and hyperinsulinemic patients. The euglycemic-hyperinsulinemic clamp is the gold standard for demonstrating insulin resistance (IR), but it is a difficult method to apply (3). Triglyceride-glucose (TyG) index and triglyceride-to-high-density lipoprotein cholesterol ratio (TG/HDL-C) have been shown to have a high correlation with euglycemic-hyperinsulinemic clamping in various studies (4). These two simple indicators are quite practical in demonstrating IR in clinical practice and studies due to their simplicity and applicability. Again, many studies have found a significant and strong relationship between CVD and the TyG index and the TG/HDL-C ratio (5–7). In patients with insulin resistance, diabetes, hypertension, and hyperlipidemia increase the risk of CVD due to endothelial dysfunction (8). In addition, the cholesterol level in the blood increases the risk of ischemic heart disease (IHD) and CVD (9, 10). Studies have shown that the ratio of total cholesterol to HDL-C (TC/HDL-C) is a determinant in predicting IHD (11, 12). CVD poses a risk in the entire population as well as in chronic hepatitis B (CHB) patients receiving antiviral treatment and those not receiving antiviral treatment. One of the main reasons for this effect is the increasing frequency of fatty liver disease (MAFLD) associated with metabolic dysfunction in the general population (13). On the other hand, studies have also shown that the antiviral drugs used cause changes in metabolic parameters. The effect of ETV on IR and the effect of TDF on cholesterol values ​​have been shown in many studies in the literature (14–20). In this study, we aimed to investigate the effects of CHB and the nucleotide analogues [NAs (ETV, TDF)] used in the treatment of CHB on IR, lipid profile, and IHD/CVD.

## 2 Materials and methods

### 2.1 Research ethics

This retrospective study was approved by the Istanbul Medipol University Non-Interventional Clinical Research Ethics Committee with the decision dated February 15, 2024, and numbered 2024\196. It was prepared considering the Declaration of Helsinki - Guidance Recommendations for Medical Practitioners in Biomedical Research Involving Human Subjects.

### 2.2 Study subject, cardiovascular disease evaluation, and participants

This study is a retrospective cohort study evaluating patients diagnosed with CHB who applied to the gastroenterohepatology outpatient clinic between June 2022 and January 2024. The anamnesis, physical examination, non-invasive laboratory biomarkers, medications used, and laboratory and imaging tests of all patients diagnosed with CHB who applied within 18 months were evaluated, and potential risk factors for CVD were evaluated. The diagnosis of a total of 16 CAD patients (n = 5) who were not using medication, (n = 5) who were using TDF, and (n = 6) who were using ETV were confirmed by coronary angiography performed by a cardiologist. Inclusion criteria for the study were; adult patients over 18 years of age; patients without liver cirrhosis; inactive CHB patients who were hepatitis B surface antigen (HBsAg) positive for 18 months; patients who were HBsAg positive for 18 months and received 0.5 mg/day ETV treatment regularly for ≥6 months; patients who were HBsAg positive for 18 months and received 245 mg/day TDF regularly for ≥6 months; and patients who underwent coronary angiography within 18 months and were diagnosed with coronary artery disease were included in the study. According to the CHB prevention guidelines (2022) (21), 145 patients with a standard CHB diagnosis and an indication for antiviral treatment were started on TDF or ETV treatment according to the Modified Knodell scoring system in liver biopsy if their histological activity index was ≥6 out of 18 or fibrosis was ≥2 out of 6. A liver biopsy was not performed, and CHB treatment was not given to 96 inactive CHB patients with a standard CHB diagnosis according to the CHB prevention guidelines but without an indication for antiviral treatment. Anti-lipemic treatment of patients with hyperlipidemia (HL) was started by the cardiologist, and patients were using either atorvastatin 20 mg/day or 40 mg/day. In addition, treatment of patients with diabetes mellitus (DM) was started by the endocrinologist, and patients were using oral antidiabetic drugs (OAD) and/or insulin treatment. This detailed information was obtained from the hospital database and recorded. The exclusion criteria from the study were patients with liver cirrhosis, patients with delta hepatitis, and patients who were not pregnant or lactating. The study was designed according to the Strengthening the Reporting of Observational Studies in Epidemiology (STROBE) guidelines. Since the study concept was retrospective, it was conducted with hospital permission and consent from the hospital database, and no consent was obtained from the patients. The questionnaire was not given to the patients.

### 2.3 Study design

To demonstrate the changes in metabolic parameters, lipid profile, and insulin resistance of CHB and drugs used in CHB treatment (ETV and TDF) with non-invasive biomarkers and to determine the possible IHD/CVD risk of these effects.

**Figure 1:**
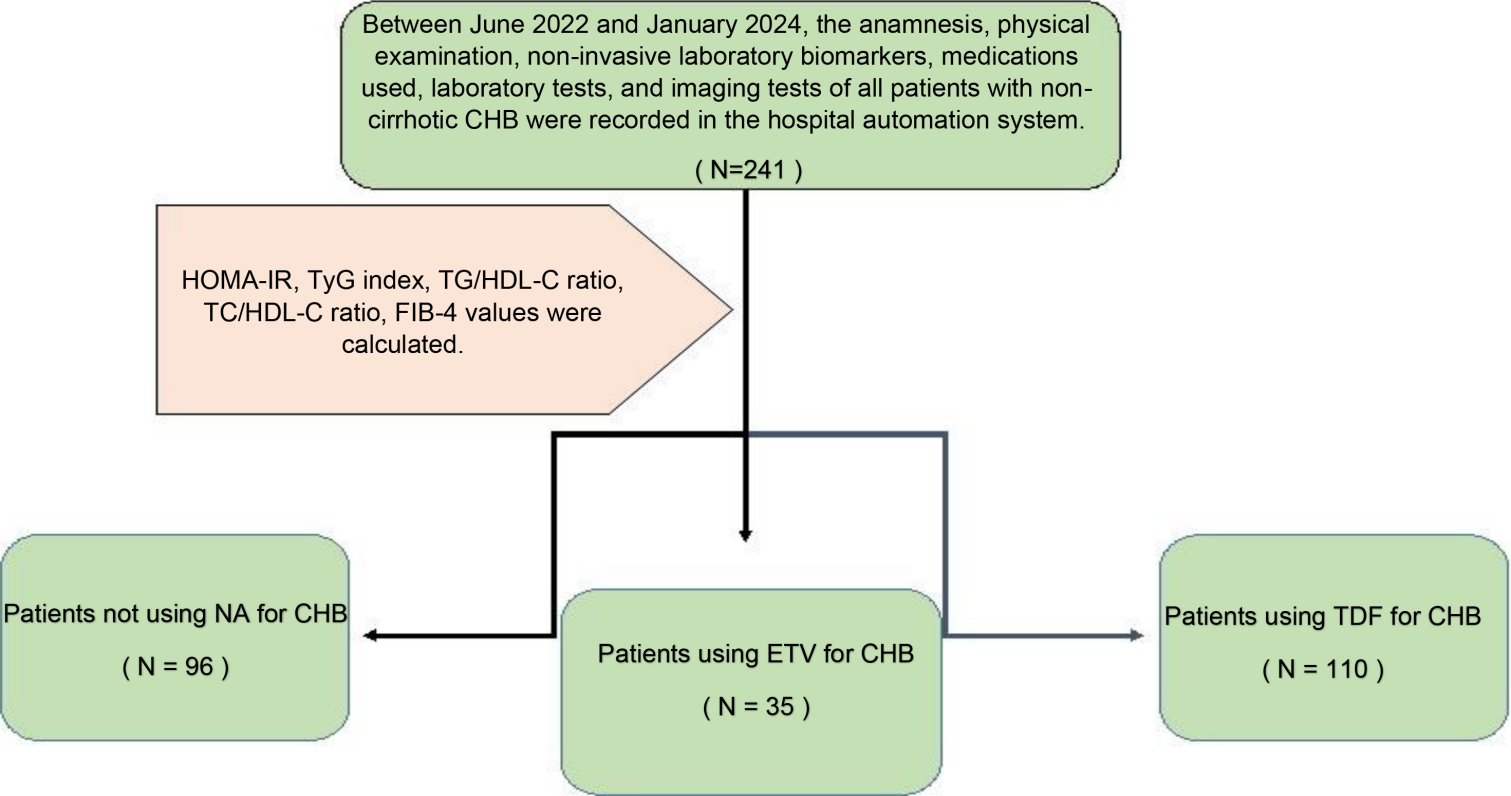
Flowchart of the participants.

This study was conducted with a total of 241 patients who applied to our gastroenterohepatology clinic, diagnosed with non-cirrhotic CHB, 96 of whom had inactive hepatitis B without hepatitis B treatment, 110 of whom received 245 mg/day TDF, and 35 of whom received 0.5 mg/day ETV treatment. Demographic characteristics of 241 non-cirrhotic CHB patients, whether they received medical treatment, which medical treatment they received, biochemical parameters, and the presence of hepatosteatosis were recorded for statistical analysis by viewing them from the hospital automation system. In the follow-up of patients diagnosed with CHB without delta agent, alanine transferase (ALT), aspartate transferase (AST), alkaline phosphatase (ALP), gamma-glutamyl transferase (GGT), hemogram, glucose, uric acid, total cholesterol (TC), high-density lipoprotein (HDL), low-density lipoprotein (LDL), triglyceride (TG), and insulin values ​​were recorded. In line with these data, IR indicators HOMA-IR, triglyceride-glucose (TyG) index, and triglyceride-to-high-density lipoprotein cholesterol (TG/HDL-C) ratio were calculated. Again, while the ratio of total cholesterol to HDL-C (TC/HDL-C), which is associated with CVD/IHD, was evaluated, the ‘4-factor fibrosis index’ (FIB-4) score, which is a non-invasive indicator of liver fibrosis, was also evaluated (**Figure 1**).

*Biomarker formulas used in the study:*

- HOMA-IR= Fasting glucose (mg/dL) X fasting insulin (IU/mL)/405,
  Insulin resistance (IR) was considered positive in patients with a HOMA score ≥2.7.
- TyG index: ln [triglycerides (mg/dL) x glucose (mg/dL)/2].
- TG/HDL-C ratio; ratio of TG (mg/dL) to HDL-C (mg/dL).
- TC/HDL-C ratio; ratio of TC (mg/dL) to HDL-C (mg/dL).
- FIB-4: Age (years)X AST (U/L)/[Platelet count (10^9^/L)×(ALT)^1\2^(U/L).

### 2.4 Statistical analysis

A G-power test was performed before starting the study. However, since the number of participants required for G-power was not sufficient, data were calculated on the patients included according to the study criteria.

The SPSS 26 (Statistical Package for the Social Sciences) program was used for statistical analysis while evaluating the findings obtained in the study. While evaluating the research data, quantitative variables were shown with mean, standard deviation, median, minimum, and maximum values, and qualitative variables were shown with descriptive statistical methods such as frequency and percentage. Shapiro Wilks test and Box Plot graphics were used to evaluate the conformity of the data to normal distribution. The one-way ANOVA test was used to compare three or more quantitative groups with normal distribution, and the Bonferroni test was used to determine the group causing the difference. The Kruskal-Wallis test was used to compare variables that did not show the normal distribution in three or more groups, and the Dunn test was used to determine the group causing the difference. The chi-square test was used to compare qualitative data. ROC curve analysis and screening tests were performed to predict ETV treatment. The results were evaluated at a 95% confidence interval, and the significance level was p<0.05.

Sensitivity: The test can identify patients among real patients.

Specificity: The test can identify healthy individuals among real healthy individuals.

Positive Predictive Value (PPV): It is the measure of the conditional probability that the subject is sick when the test gives a positive (sick) result.

Negative Predictive Value (NPV): It is the probability that the subject is actually healthy when the test gives a negative (healthy) result.

## 3 Results

**Figure 2:**
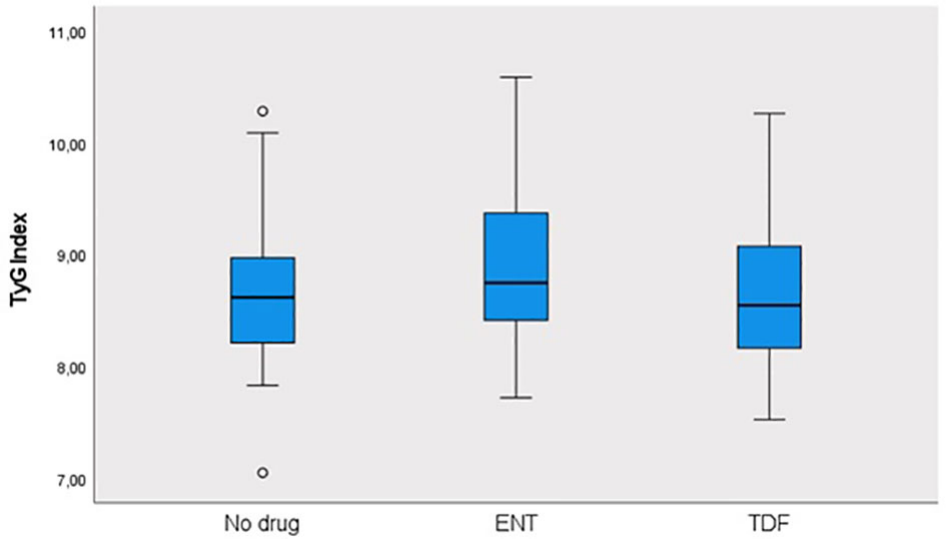
Distribution of TyG index in groups.

**Figure 3:**
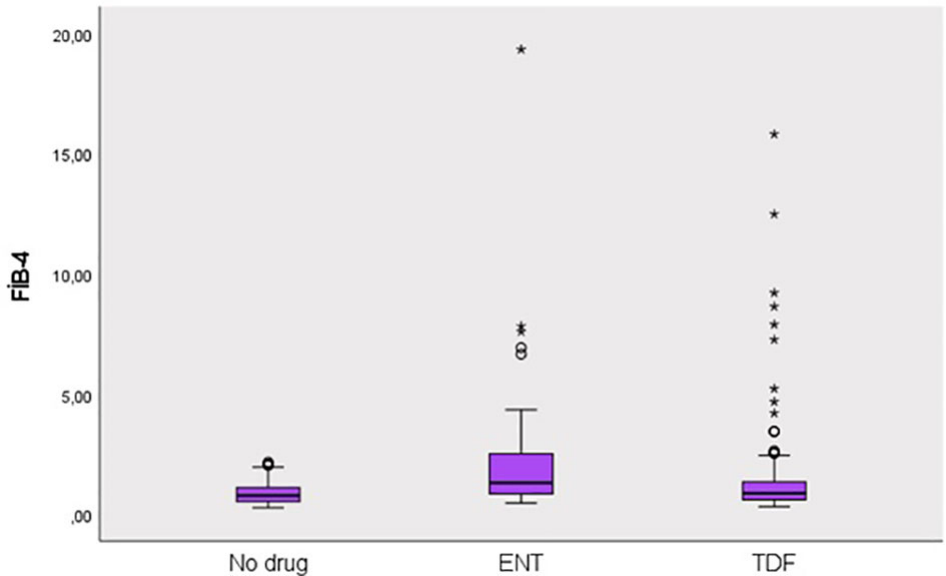
Distribution of FIB-4 score in groups. Round (Extreme value): Outside 1.5 times the interquartile range above the upper quartile and below the lower quartile (Q1 – 1.5 * IQR or Q3 + 1.5 * IQR). Asterisk (Very extreme value): Outside 3 times the interquartile range above the upper quartile and below the lower quartile (Q1 – 3 * IQR or Q3 + 3* IQR). IQR is the size of the box.

This study was conducted in our center between June 2022 and January 2024 with a total of 241 patients; 57.3% (n = 138) were male and 42.7% (n = 103) were female. The ages of the patients ranged from 18 to 80, with a mean of 46.1 ± 14.2. 12.4% (n = 30) of the patients participating in the study had a known diagnosis of diabetes mellitus (DM). 10.8% (n = 26) of these patients were using oral antidiabetic (OAD) drugs, while 5.4% (n = 13) were using insulin. 6.6% (n = 16) of the patients participating in the study had a known diagnosis of coronary artery disease (CAD). 12.4% (n = 30) of the patients were receiving hyperlipidemia (HL) treatment. The patients’ TyG index scores ranged from 7 to 10.6, with a mean of 8.67 ± 0.60. The FIB-4 scores of the patients included in the study ranged from 0.3 to 19.3, with a mean of 1.48 ± 2.18. There was no hepatosteatosis on abdominal ultrasound in 80.9% (n = 195) of the patients. However, 16.6% (n = 40) had grade 1 hepatosteatosis, 2.1% (n = 5) had grade 2 hepatosteatosis, and 0.4% (n = 1) had grade 3 hepatosteatosis. Hepatitis B e antigen (HBeAg) positivity was observed in 10.4% (n = 25) of the patients included in the study. While 39.8% (n = 96) of the patients did not use antiviral drugs, 14.5% (n = 35) used ETV, and 45.6% (n = 110) used TDF (**Table 1**). The ages of the patients using ETV were higher than those not using drugs and using TDF (p = 0.001; p = 0.003; p<0.01). The rate of DM in the ETV group was higher than in those not using drugs and using TDF. A significant difference was found between OAD and insulin use in patients with CHB according to the drug use status [respectively (p = 0.006; p<0.01), (p = 0.024; p<0.05)]. The rate of OAD and insulin use was higher in patients using ETV than in patients not using drugs and using TDF. The incidence of CAD was higher in patients using ETV than in patients not using medication and using TDF (p = 0.026; p<0.05). The TyG index of patients using ETV was higher than in patients not using medication and using TDF (p = 0.035; p<0.05) (**Figure 2**). The FIB-4 scores of patients not using medication were lower than in patients using ETV and TDF (p = 0.001; p = 0.043; p<0.05). Again, the FIB-4 scores of patients using ETV were higher than in patients not using medication and using TDF (p = 0.039; p<0.05) (**Figure 3**). The presence of HBeAg in patients with CHB showed a statistically significant difference between the groups (p = 0.001; p<0.01). HBeAg positivity was lower in patients not using medication than in patients using TDF and ETV (**Table 2**). The FBG values ​​of patients using ETV were higher than those using no medication and those using TDF (p = 0.001; p = 0.002; p<0.01). The HDL values ​​of patients using no medication were higher than those using ETV and TDF (p = 0.003; p = 0.004; p = 0.01). The total cholesterol values ​​of patients using no medication were higher than those using TDF (p = 0.008; p=0.01) (**Table 3**).

**Table 1:** Demographic and clinical characteristics of patients diagnosed with CHB.

		n (%)
**Sex**	**Male**	138 (57.3)
	**Female**	103 (42.7)
**Age**	*Mean ± Sd*	46.1 ± 14.2
	*Median (Min-Max)*	46 (18–80)
**Diabetes Mellitus**	**Absent**	211 (87.6)
	**Existent**	30 (12.4)
**Oral anti-diabetic** **drug use**	**Absent**	215 (89.2)
	**Existent**	26 (10.8)
**Insulin use**	**Absent**	228 (94.6)
	**Existent**	13 (5.4)
**Coronary Artery Disease**	**Absent**	225 (93.4)
	**Existent**	16 (6.6)
**Hyperlipidemia treatment**	**Absent**	211 (87.6)
	**Existent**	30 (12.4)
**TyG Index**	*Mean ± Sd*	8.67 ± 0.60
	*Median (Min-Max)*	8.6 (7-10.6)
**FIB-4**	*Mean ± Sd*	1.48 ± 2.18
	*Median (Min-Max)*	0.9 (0.3-19.3)
**Ultrasound Hepatosteatosis**	**Grade 0**	195 (80.9)
	**Grade 1**	40 (16.6)
	**Grade 2**	5 (2.1)
	**Grade 3**	1 (0.4)
**HBeAg**	**Absent**	215 (89.6)
	**Existent**	25 (10.4)
**Medical treatment Status**	**No drug**	96 (39.8)
	**ENT**	35 (14.5)
	**TDF**	110 (45.6)

TyG index, Triglyceride-glucose index; FIB-4 score, 4-factor fibrosis index; HBeAg, Hepatitis B e-Antigen; ENT, entecavir; TDF, tenofovir disoproxil fumarate.

**Table 2:** Comparison of descriptive characteristics by drug use status.

		No drug (n=96)	ENT (n=35)	TDF (n=110)	*p*
**Sex**	**Male**	49 (51.0)	24 (68.6)	65 (59.1)	***^a^0.183***
	**Female**	47 (49.0)	11 (31.4)	45 (40.9)	
**Age**	*Mean ± Sd*	43.5 ± 14.6	54.5 ± 13.7	45.7 ± 13.0	^b^0.001**
	*Median (Min-Max)*	42 (18–75)	56 (29–80)	45.5 (21–76)	
**Diabetes Mellitus**	**Absent**	89 (92.7)	24 (68.6)	98 (89.1)	^a^0.001**
	**Existent**	7 (7.3)	11 (31.4)	12 (10.9)	
**Oral anti-diabetic drug use**	**Absent**	90 (93.8)	26 (74.3)	99 (90.0)	^a^0.006**
	**Existent**	6 (6.3)	9 (25.7)	11 (10.0)	
**Insulin use**	**Absent**	94 (97.9)	30 (85.7)	104 (94.5)	^a^0.024*
	**Existent**	2 (2.1)	5 (14.3)	6 (5.5)	
**Coronary Artery Disease**	**Absent**	91 (94.8)	29 (82.9)	105 (95.5)	^a^0.026*
	**Existent**	5 (5.2)	6 (17.1)	5 (4.5)	
**Hyperlipidemia treatment**	**Absent**	86 (89.6)	27 (77.1)	98 (89.1)	***^a^0.130***
	**Existent**	10 (10.4)	8 (22.9)	12 (10.9)	
**TyG Index**	*Mean ± Sd*	8.64 ± 0.54	8.91 ± 0.73	8.61 ± 0.59	^b^0.035*
	*Median (Min-Max)*	8.6 (7-10.3)	8.7 (7.7-10.6)	8.5 (7.5-10.3)	
**FIB-4**	*Mean ± Sd*	0.89 ± 0.46	2.64 ± 3.55	1.62 ± 2.36	^b^0.001**
	*Median (Min-Max)*	0.8 (0.3-2.2)	1.3 (0.5-19.3)	0.9 (0.3-15.8)	
**Ultrasound Hepatosteatosis**	**Absent**	79 (82.3)	26 (74.3)	90 (81.8)	***^a^0.582***
	**Grade 1-2-3**	17 (17.7)	9 (25.7)	20 (18.2)	
**HBeAg**	**Absent**	94 (97.9)	31 (91.2)	90 (81.8)	^a^0.001**
	**Existent**	2 (2.1)	3 (8.8)	20 (18.2)	

^a^Pearson Chi-Square Test ^b^One-Way ANOVA Test & Bonferroni Test **p<0.01 *p<0.05.

The ages of patients using ETV are higher than those not using medication and using TDF (p=0.001; p=0.003; p<0.01). The rate of DM in patients using ETV is higher than that of patients not using medication and using TDF (p=0.001; p<0.01). The OAD use of patients using ETV is higher than that of patients not using medication and using TDF (p=0.006; p<0.01). The insulin use of patients using ETV is higher than that of patients not using medication and using TDF (p=0.024; p<0.05). The rate of CAD in patients using ETV is higher than that of patients not using medication and using TDF (p=0.026; p<0.05). The TyG index in patients using ETV is higher than that of patients using TDF (p=0.035; p<0.05). While the FIB-4 score of patients not using medication was lower, the FIB-4 score of patients using ETV was higher than that of patients using TDF (p=0.001; p<0.01). HBeAg positivity was higher in patients using TDF (p=0.001; p<0.01).

**Table 3:** Comparison of laboratory values, diagnostic metabolic and cardiovascular markers according to drug use status.

		Total	No drug (n=96)	ENT (n=35)	TDF (n=110)	*p*
**ALT**	*Mean ± Sd*	51.57 ± 159.69	25.98 ± 19.14	54.5 ± 107.81	72.97 ± 226.19	***^c^0.108***
	*Median (Min-Max)*	23 (8–1928)	20 (8–123)	21 (8–580)	26 (9–1928)	
**AST**	*Mean ± Sd*	41.63 ± 107.72	21.64 ± 7.77	52.88 ± 87.01	55.5 ± 150.17	***^c^0.063***
	*Median (Min-Max)*	21 (7–1227)	20 (11–49)	20 (7–328)	22 (11–1227)	
**ALP**	*Mean ± Sd*	91.92 ± 51.51	87.19 ± 51.96	92.43 ± 40.78	95.89 ± 54.18	***^b^0.482***
	*Median (Min-Max)*	81 (46–542)	79.5 (46–542)	82 (53–235)	83 (46–459)	
**GGT**	*Mean ± Sd*	42.32 ± 104.19	37.22 ± 139.70	51.26 ± 79.41	43.94 ± 69.82	***^c^0.775***
	*Median (Min-Max)*	19 (6–1365)	16.5 (6–1365)	21 (9–358)	21 (7–513)	
**Fasting Blood Sugar**	*Mean ± Sd*	105.06 ± 40.78	97.85 ± 19.52	130.35 ± 61.36	103.30 ± 43.60	^b^0.001**
	*Median (Min-Max)*	95 (60–449)	94 (60–183)	103 (77–303)	94.8 (69–449)	
**Uric Acid**	*Mean ± Sd*	4.59 ± 1.29	4.53 ± 1.21	4.69 ± 1.47	4.60 ± 1.30	***^b^0.809***
	*Median (Min-Max)*	4.5 (1-9.4)	4.6 (1-7.9)	4.5 (2.2-9.4)	4.5 (1.9-9)	
**LDL**	*Mean ± Sd*	93.9 ± 29.85	97.82 ± 28.08	93.95 ± 28.23	90.46 ± 31.63	***^b^0.211***
	*Median (Min-Max)*	92.4 (27-238.1)	95 (54.3-212.5)	93.7 (51.4-156.3)	91.6 (27-238.1)	
**HDL**	*Mean ± Sd*	46.83 ± 14.19	51.03 ± 14.69	42.00 ± 11.73	44.70 ± 13.60	^b^0.001**
	*Median (Min-Max)*	45.8 (9.3-129.8)	50.8 (26.9-129.8)	41.7 (9.3-67.9)	43.8 (13-88.6)	
**Total Cholesterol (TC)**	*Mean ± Sd*	166.64 ± 34.28	174.81 ± 32.61	163.24 ± 33.84	160.59 ± 34.69	^b^0.009**
	*Median (Min-Max)*	167 (81–295)	171 (106–295)	163 (104–231)	159.5 (81–295)	
**Triglyceride (TG)**	*Mean ± Sd*	130.35 ± 72.29	132.01 ± 72.3	136.05 ± 66.75	127.09 ± 74.4	***^b^0.784***
	*Median (Min-Max)*	112.7 (28.3-566.2)	117 (28.3-566.2)	114 (53.4-334.4)	108.3 (42.3-549)	
**Insulin**	*Mean ± Sd*	21.01 ± 24.08	17.85 ± 20.42	21.18 ± 22.28	23.72 ± 27.24	***^b^0.219***
	*Median (Min-Max)*	11.7 (1.6-151.6)	10.2 (3-151.6)	11.8 (2.9-111.7)	12.2 (1.6-142.5)	
**HBV DNA**	*Mean ± Sd*	1.52 ± 2.14	1.96 ± 1.79	1.00 ± 1.99	1.30 ± 2.39	^c^0.027*
	*Median (Min-Max)*	0 (0–8)	2.6 (0–8)	0 (0-6.2)	0 (0–8)	
**HgA1c**	*Mean ± Sd*	5.96 ± 3.56	6.08 ± 5.39	6.39 ± 1.98	5.71 ± 1.10	***^c^0.564***
	*Median (Min-Max)*	5.5 (4–58)	5.4 (4.4-58)	5.6 (4-12.5)	5.5 (4.1-13.5)	
**HOMA-IR**	*Mean ± Sd*	6.09 ± 9.21	4.80 ± 7.43	8.15 ± 13.42	6.56 ± 8.90	***^c^0.142***
	*Median (Min-Max)*	2.8 (0.4-77.5)	2.4 (0.6-63.6)	5.1 (0.6-77.5)	3.2 (0.4-54.5)	
**TG/HDL-C**	*Mean ± Sd*	3.24 ± 2.49	2.90 ± 2.14	3.69 ± 2.46	3.38 ± 2.75	***^b^0.195***
	*Median (Min-Max)*	2.5 (0.7-16.8)	2.4 (0.8-16.8)	2.7 (1.1-11.5)	2.5 (0.7-15.7)	
**TC/HDL-C**	*Mean ± Sd*	3.88 ± 1.52	3.64 ± 1.05	4.34 ± 2.24	3.95 ± 1.56	***^b^0.052***
	*Median (Min-Max)*	3.6 (1.5-15.1)	3.5 (1.8-6.4)	4.1 (2.3-15.1)	3.7 (1.5-10.1)	

^b^One-Way ANOVA Test & Bonferroni Test ^c^Kruskal Wallis Test & Dunn-Bonferroni Test **p<0.01 *p<0.05.

ALT, alanine transferase; AST, aspartate transferase; ALP, alkaline phosphatase; GGT, gamma-glutamyl transferase; TC, total cholesterol; HDL, high-density lipoprotein; LDL, low-density lipoprotein; TG, triglyceride; TyG index, triglyceride-glucose index; TG/HDL-C, ratio of triglyceride to high-density lipoprotein cholesterol; TC/HDL-C, total cholesterol (TC) to HDL-C ratio.

Fasting blood glucose values of patients using ETV were higher than patients not using medication and using TDF (p=0.001; p=0.002; p<0.01). HDL values of patients not using medication were higher than patients using ETV and TDF (p=0.003; p=0.004; p=0.01). The total cholesterol values of patients not using medication were higher than patients using TDF (p=0.008; p=0.01). PLT values of patients using ETV were higher than patients not using medication and using TDF (p=0.001; p=0.001; p=0.01). HBV DNA values of patients not using medication were higher than patients using TDF and ETV (p=0.027; p<0.05).

**Figure 4:**
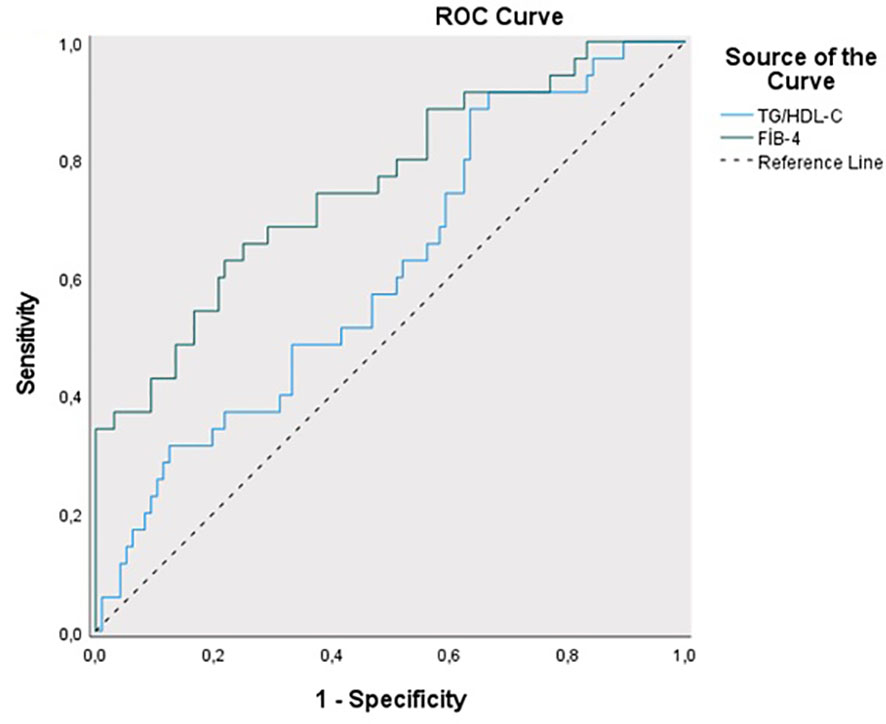
ROC curve of TG/HDL-C and FIB-4 score in predicting the presence of ENT treatment.

The sensitivity for predicting ETV treatment was 88.57%, and the specificity was 55.42% for the TG/HDL-C ratio cut-off value of 1.82 (PPV = 53.30%, NPV = 89.50%). The area under the obtained ROC curve was 61.2% with a standard deviation of 5.4% (**Figure 4**). A statistically significant relationship was found between the TG/HDL-C ratio and the cut-off value of 1.82 in predicting ETV treatment (p = 0.048; p<0.05). It was observed that the probability of detecting ETV treatment was 4.250 times higher in cases with a TG/HDL-C ratio ≥1.82. The odds ratio for TG/HDL-C ratio was 4.250 (95% CI: 1.384–13.054). For the cut-off value of 1.15 for the FIB-4 score in predicting ETV treatment, sensitivity was 62.86% and specificity was 77.08% (PPV = 50.00%, NPV = 85.10%). The area under the obtained ROC curve was 75.8% with a standard deviation of 4.9%. A statistically significant relationship was found between the FIB-4 score and the cut-off value of 1.15 in predicting ETV treatment (p = 0.048; p<0.05). It was observed that the probability of detecting ETV treatment was 5.692 times higher in cases with a FIB-4 score ≥ 1.15. The odds ratio for the FIB-4 score was 5.692 (95% CI: 2.471-13.115). No statistically significant relationship was found between the TyG index and TC/HDL-C ratios in predicting ETV treatment (p > 0.05) (**Table 4**).

**Table 4:** Diagnostic screening tests and ROC curve results for TyG index, TG/HDL-C, T.Cholesterol/HDL-C ratio and FIB-4 scores in predicting ENT treatment.

	Diagnostic Scan					ROC Curve		*p*
	Cut off	Sensitivity	Specificity	Positive Predictive Value	Negative Predictive Value	Area	95% Confidence Interval	
**TyG Index**	***≥9.34***	28.57	91.67	55.60	77.90	0.599	0.485-0.714	***0.082***
**TG/HDL-C**	***≥1.82***	88.57	55.42	53.30	89.50	0.612	0.506-0.718	0.048*
**TC/HDL-C**	***≥4.04***	51.43	66.67	56.00	79.00	0.591	0.478-0.704	***0.111***
**FIB-4**	***≥1.15***	62.86	77.08	50.00	85.10	0.758	0.662-0.854	0.001**

*p<0.05 **p<0.01.

For the cut-off value of 1.82 for TG/HDL-C ratio in predicting ETV treatment, sensitivity was 88.57% and specificity was 55.42% (PPV=53.30%, NPV=89.50%). The area under the obtained ROC curve was 61.2% with a standard deviation of 5.4%. There was a statistically significant relationship between the cut-off value of 1.82 for the TG/HDL-C ratio in predicting ETV treatment (p=0.048; p<0.05). For the cut-off value of 1.15 for the FIB-4 score in predicting ETV treatment, sensitivity was 62.86% and specificity was 77.08% (PPV=50.00%, NPV=85.10%). The area under the obtained ROC curve was 75.8% with a standard deviation of 4.9%. A statistically significant relationship was found between the FIB-4 score and the cut-off value of 1.15 in predicting ETV treatment (p=0.048; p<0.05). There was no statistically significant relationship between the TyG index and TC/HDL-C ratios in predicting ETV treatment (p>0.05).

## 4 Discussion

Many studies have been conducted on the relationship between NAs (ETV, TDF) used in the treatment of CHB and metabolic syndrome in the last decade (14–20). Many recent studies have shown that the relationship between TyG index and IHD and CVD is strong (23–25). In the Kailuan study by Liu et al., a high correlation was found between the TyG index and CVD in 96,541 participants who were followed for an average of 10.33 years. The striking point in this study is that the potential relationship between the TyG index and CVD was concluded based on data obtained from the general population rather than from certain high-risk groups (26). Again, in a Chinese cohort of the general population, the TyG index was calculated for 62,443 patients without CVD, and it was shown that the change in this index could predict the risk of CVD (22). A cohort study of 10,761 participants showed that individuals at risk of MAFLD may be present when the TyG index is at a cut-off value of 8.5 (27). Another cohort study of the general population found a positive correlation between MAFLD and the TyG index (28). It has been proven that the TyG index can predict both MAFLD and CVD non-invasively. However, there is no study predicting the effect of changes in metabolic events on CVD in patients diagnosed with CHB. Therefore, this relationship was investigated in this study. In our study, we found the mean TyG index to be 8.91 ± 0.73 (p=0.035) in patients using ETV. In addition, it was determined that the TG/HDL-C cut-off value could be ≥1.82 in predicting the use of ETV in patients (sensitivity 88.57%; specificity 55.42%; PPV = 53.30%; NPV = 89.50%) (p = 0.048; p<0.05). We found that the probability of receiving ETV treatment was 4.250 times higher at the cut-off value of TG/HDL-C ratio ≥1.82. The odds ratio for the TG/HDL-C ratio was 4.250 (95% CI: 1.384–13.054). This situation suggests that more belief should be given to the idea that CHB and the NAs used in its treatment may be related to CVD with a multi-factorial etiology. In the study of Kosmas et al., the TG/HDL-C ratio was shown to be an excellent biomarker in predicting IR and CVD risk (29). Each 1 SD increase in the log-transformed values ​​of the TyG index and TG/HDL-C ratio was associated with an 8% and 12% increase in CVD risk, respectively (12). In our study, among the patients with CHB who used NAs (ETV, TDF) or did not use NAs, the significant elevations in both TyG index and TG/HDL-C ratio biomarkers in patients who used ETV may indicate an increase in CVD risk. This evaluation may help us in the evaluation of patients in the risk group, in their follow-up, and in switching to different medical treatments if necessary.

One of the non-invasive tests that is effective in showing liver fibrosis in CHB patients is the FIB-4 score (30). The FIB-4 score of patients using ETV was 2.64 ± 3.55, and we found that liver fibrosis was higher than other patient groups (p = 0.001). We found that the probability of predicting ETV use was 5.692 times higher in cases with a cut-off value of FIB-4 score ≥1.15. We assume that the additional contribution of IR and steatotic liver disease associated with metabolic dysfunction may be the reason for this high FIB-4 score. We also found that NAs, especially the ETV molecule, used in patients diagnosed with CHB increased HOMA-IR, similar to the literature (14). Studies have shown that IR has great importance in the pathophysiology of atherosclerosis, IHD, and CVD. Studies have shown that TC/HDL-C is another biomarker for predicting IHD (31). In our study, we did not see a significant change in the TC/HDL-C ratio between patients with CHB who received medical treatment and those who did not. However, we found that the TC/HDL-C ratio was relatively higher in those who received ETV treatment, although not statistically significant, with an average of 4.34 ± 2.24. Another issue that is striking in our study and similar to the literature is that patients using NAs (ETV, TDF) had lower total cholesterol levels than patients who did not use medication (14, 18). Again, the HDL values ​​of patients using NAs (ETV, TDF) were lower than patients who did not use medication. Although low TC levels seem to be good in terms of CVD, the effect of low HDL on CVD is unclear. We know that HDL cholesterol, unlike LDL cholesterol, plays an anti-atherogenic role by transporting cholesterol from the periphery to the liver (32). Another issue that draws attention in our study is that in patients using ETV due to CHB diagnosis, FBG, and HgA1c levels were higher than in patients using TDF or not using medication. Again, in our study, the presence of DM, OAD, and insulin use was higher in patients using ETV. When we evaluated the IR in CHB patients using ETV with the TyG index, which shows IR independent of DM diagnosis, in order to show that IR increased, it confirmed that ETV increased IR. In our study, we found the TyG index higher in patients using ETV than in patients not using medication or using TDF. We know that studies have shown that the Tyg index obtained from triglycerides and FBG levels correlates with HOMA-IR and the hyperinsulinemic-euglycemic clamp test (4, 33). In addition to being a marker of CVD risk, the TyG index has also been shown to be a reliable indicator of IR in many studies (27, 32, 33).

### Strengths and limitations of the study

The strength of this study is that we believe that the results have important clinical implications. The short-term and long-term changes in lipid profiles in patients using and not using NAs due to CHB and the development of IR are inevitable, resulting in end-organ damage. Changes in metabolic parameters are associated with CVD. Although studies have shown that metabolic changes may occur in patients diagnosed with CHB, this is the first study to investigate the possibility of developing CVD. We have shown that IR and CVD risk may develop in the group of patients using ETV due to CHB. We emphasized that patients diagnosed with CHB should be evaluated holistically not only in terms of hepatology but also in terms of metabolism and CVD. With this study, we emphasized that the patient should be evaluated from a broader perspective. Although changes in metabolic events are emphasized in the literature, the fact that the effects of end-organ damage are not clearly emphasized led us to conduct this study. However, this study had several limitations on the other hand. The most important limiting factors of our study are that it is a retrospective study, a cross-sectional evaluation is conducted, the height, weight, and body mass index of our patients cannot be evaluated, and our sample size is not large enough. Secondly, TC/HDL-C, TyG index, and TG/HDL-C ratio were determined from a single blood sample, therefore, we could not evaluate the effect of their changes on CVD over time. Thirdly, cirrhotic patients due to CHB were not included in the study. Although we did not want to show the metabolic changes that cirrhosis will cause here, the fact that cirrhotic patients were not included in the study was a limiting situation. In addition, the fact that clinicians have been provoked to choose ETV as NA for CHB treatment in patient groups with high risk for CVD (advanced age, presence of comorbid diseases, especially chronic renal failure, osteoporosis, hypophosphatemia) suggests that ETV may increase the possible CVD risk. We know that TDF has complications such as possible hypophosphatemia, osteoporotic bone changes, and proximal tubule dysfunction. Although this bias in the preference for ETV seems to be more significant in terms of CVD, the effect of TDF on CVD is not clear. Therefore, despite its various limitations, this retrospective study is quite important for the beginning of multicenter, multiethnic, prospective randomized cohort studies.

## 5 Conclusion

A relationship was observed between biomarkers used to predict IR and CVD risk, such as TyG index and TG/HDL-C ratio, in patients receiving and not receiving CHB treatment. The group with high levels of these two markers and a high potential for developing CVD was patients receiving ETV treatment. In this first study in the literature showing the relationship between CHB and CVD, we found that the risk of CVD was relatively increased in patients receiving ETV.

## Data Availability

The original contributions presented in the study are included in the article/supplementary material. Further inquiries can be directed to the corresponding author.
